# Could β-Lactam Antibiotics Block Humoral Immunity?

**DOI:** 10.3389/fimmu.2021.680146

**Published:** 2021-09-15

**Authors:** Cléa Melenotte, Pierre Pontarotti, Lucile Pinault, Jean-Louis Mège, Christian Devaux, Didier Raoult

**Affiliations:** ^1^Aix-Marseille Univ, Institut de Recherche et Développement (IRD), Assistance Publique des Hpitaux de Marseille (APHM), Microbes, Evolution, Phylogénie et Infection (MEPHI), Marseille, France; ^2^Institut Hospitalo-Universitaire (IHU)-Méditerranée Infection, Marseille, France; ^3^Centre National de la Recherche Scientifique (CNRS), Marseille, France

**Keywords:** ARTEMIS, SNM1, human metallo β-lactamase, metallo β-lactamase, β-lactam antibiotics, cephalosporin, immunity, V(D)J recombination

## Abstract

It has been reported that treatment with β-lactam antibiotics induces leukopenia and candidemia, worsens the clinical response to anticancer immunotherapy and decreases immune response to vaccination. β-lactamases can cleave β-lactam antibiotics by blocking their activity. Two distincts superfamilies of β-lactamases are described, the serine β-lactamases and the zinc ion dependent metallo-β-lactamases. In human, 18 metallo-β-lactamases encoding genes (hMBLs) have been identified. While the physiological role of most of them remains unknown, it is well established that the SNM1A, B and C proteins are involved in DNA repair. The SNM1C/Artemis protein is precisely associated in the V(D)J segments rearrangement, that leads to immunoglobulin (Ig) and T-cell receptor variable regions, which have a crucial role in the immune response. Thus in humans, SNM1C/Artemis mutation is associated with severe combined immunodeficiency characterized by hypogammaglobulinemia deficient cellular immunity and opportunistic infections. While catalytic site of hMBLs and especially that of the SNM1 family is highly conserved*, in vitro* studies showed that some β-lactam antibiotics, and precisely third generation of cephalosporin and ampicillin, inhibit the metallo-β-lactamase proteins SNM1A & B and the SNM1C/Artemis protein complex. By analogy, the question arises as to whether β-lactam antibiotics can block the SNM1C/Artemis protein in humans inducing transient immunodeficiency. We reviewed here the literature data supporting this hypothesis based on *in silico*, *in vitro* and *in vivo* evidences. Understanding the impact of β-lactam antibiotics on the immune cell will offer new therapeutic clues and new clinical approaches in oncology, immunology, and infectious diseases.

## Introduction

β-lactam antibiotics remain the cornerstone of the antibacterial arsenal, being the most prescribed and the most important class of antibiotics in terms of sales (1–3). In clinical practice, β-lactam antibiotics are increasingly used to treat pulmonary, urinary, skin, osteo-articular and cardio-vascular infections (2, 4). However, parenteral use of β-lactam antibiotics was associated, in human, with an increased risk of complications such as candidemia, especially in the presence of catheters (5). In addition, its utilization prior to anticancer immunotherapy was associated with a worse clinical response (6–8). Nevertheless the direct effect of β-lactam antibiotics on immune cells remains poorly understood (9–15).

β-lactam antibiotics may have multiple activities. First of all, it works by inhibiting the bacterial peptidoglycan cell wall synthesis (16). β-lactamases enzymes hydrolyse the β-lactam antibiotics and may also have different nuclease and hydrolases activities (17–24). Two mechanistically distincts superfamilies of β-lactamases with two distincts ancestors are described, the nucleophilic serine β-lactamases (class A/C/D); and the zinc ion dependent metallo-β-lactamases (class B) (17, 25–27). In human, 18 metallo-β-lactamases (hMBLs) enzymes have been identified (**Table 1**) (28). While the physiological enzymatic role of most of them remains unknown, mutation of some hMBLs has been identified as linked to clinical disease (**Table 1**) (28–31). This is the case for the SNM1C/Artemis mutation that induces severe combined immunodeficiency characterized by hypersensitivity to ϒ rays irradiation and severe opportunistic infections (see next chapter) (28).

**Table 1:** Human metallo beta lactamases function (28).

18 hMBLs	hMBL protein	Gene and chromosome location	Enzymatic activity	Function/Proposed Function	Linked Diseases
1	HAGH (1XM8)	3029; 16p13.3	Hydrolase or Glyoxalase II	Glutathione biosynthetic process.	None assigned
2	HAGHL	84264; 16p13.3	Hydrolase or Glyoxalase II	Unknown	None assigned
3	ETHE1 (4CHL)	23474; 19q13.31	Glyoxalase II	Glutathione metabolic process. Hydrogen sulfide metabolic process	Ethylmalonic encephalopathy
4	LACTB2 (4AD9)	51110; 8q13.3	Glyoxalase II	Endoribonuclease activity on ssRNA	None assigned
				Mitochondrial cell death turnover	
5	MBLAC1	255374/153364; 7q22.1/5q14.3	Glyoxalase II	Cell cycle progression (S phase)	None assigned
6	MBLAC2	255374/153364; 7q22.1/5q14.3	Glyoxalase II	Penicillin degradation	None assigned
				B-cell exome biogenesis	
7	SNM1A (4B87)	9937; 10q25.1	Exonuclease	Cell cycle, DNA damage/DNA crosslink repair	Mutations: impaired nuclear focus formation, reduced interaction with PIAS and increased sensitivity to cisplatin
8	PNKD	25953; 2q35	Glyoxalase II	Unknown	Dystonia type 8
					Tumor cell proliferation (PNKD2)
9	SNM1B (5AHO)	64858; 1p13.2	Exonuclease	DNA damage/DNA crosslink repair	Hoyeraal–Hreidarsson Syndrome
10	SNM1C	64421; 10p13	Exo & endonuclease	Adaptive immunity; DNA damage, recombination, repair/DNA crosslink repair	Severe combined immunodeficiency autosomal recessive T-cell-negative/B cell-negative/NKcell-positive with sensitivity to ionizing radiation (RSSCID), Severe combined immunodeficiency Athabaskan type (SCIDA),Omenn syndrome (OS)
11	ELAC1 (3ZWF)	55520; 18q21	RNase	tRNA processing	None assigned
12	ELAC2	60528; 17p11.2	RNase	tRNA processing	Prostate cancer, hereditary, 2 (HPC2)
13	CPSF73 (2I7T)	51692; 2p25.1	Endonuclease	mRNA processing	None assigned
14	CPSF73L	54973; 1p36.33	Integrator complex	pre-snRNA processing	None assigned
			Hydrolase		
15	CPSF100	53981; 14q31.1	Unknown	mRNA processing	None assigned
16	INTS9	55756; 8p21.1	Integrator complex	snRNA processing	None assigned
			Hydrolase		
17	NAPE-PLD (4QN9)	222236; 7q22.1	Catalyse hydrolysis of NAPE	Lipid/Phospholipid degradation, metabolism	None assigned
18	CMAHP	8418; 6p21.32	Unknown	Unknown	None assigned

PIAS, Protein inhibitor of Activated STAT; NK, Natural killer; RS-SCID, Radiosensitive severe combined immunodeficiency; SCIDA,severe combined immunodeficiency disease (SCID) occuring among Athabascan-speaking Native Americans (SCIDA) LACT2B, β-lactamase-like protein 2; ELAC1, Zinc phopshodiesterase ELAC protein 1; ELAC2, Zinc phopshodiesterase ELAC protein 2 tRNA, transfert RNA; mRNA, messenger RNA; snRNA, small nuclear RNA; MBLAC1& 2, MBL domain containing protein 1 and 2CPSF73, Cleavage and polyadenylation specificity factor 73; CPSF73L, Cleavage and polyadenylation specificity factor 73 like; CPSF100, Cleavage and polyadenylation specificity factor 100; INTS9, Integrator complex subunit; NAPE-PLD (4QN9), N-acyl-phophatidylethanolamine-hydrolizing phospholipase-D; CMAHP, CMP-N-acetylneuraminic acid hydroxylase pseudogene.

Some β-lactam antibiotics showed direct effect on hMBLs. Cephalosporins were identified as competitive inhibitors of the hMBLs SNM1A, B and C nuclease (32). Ampicillin and cephalosporin, β-lactamin antibiotics, can *in vitro* inhibit the SNMIC/Artemis protein (33, 34). By analogy, this raises the question whether β-lactam antibiotics can induce reversible humoral deficiency in humans by transiently blocking the SNM1C/Artemis nuclease activity. As the conserved catalytic site is highly similar in SNM1A, B and C proteins, we postulate that the β-lactam antibiotics would be capable of inhibiting the SNM1C/Artemis protein leading to a transient immune deficit (humoral) (33, 35). Here, after introducing the SNM1C/Artemis protein functionality and implications, we will examine the *in silico*, *in vivo* and *in vitro* evidences to support our hypothesis. We searched in Medline and Google scholar for references with no language restrictions and no time limitations nor status. We performed a docking analysis to evaluate *in silico* the cephalosporin affinity with the SNM1C/Artemis catalytic site.

## The hMBLs SNM1C/Artemis Protein Functionality, Clinical Implications and Catalytic Site

The SNM1-family proteins, that belongs to the hMBLs, is characterized by a conserved catalytic site with nuclease activity implicated in the maintenance of the genome stability (36). SNM1 is a member of the SNM1 (or PSO2) gene family that encodes proteins involved in DNA processing, DNA metabolism and cell cycle regulation. Whereas SNM1A and B contribute to the intercross linked repair, SNM1C/Artemis contributes to the double-strand break repair. Indeed, SNM1C/Artemis possesses an exonuclease activity *in vitro* and when complexed and phosphorylated by DNA PKcs, SNM1C/Artemis’ specificity switched to an endonuclease activity, which is involved in the V(J) and V(D)J segments rearrangement (37).

### The hMBLs SNM1C/Artemis Protein and Its Catalytic Site

**Figure 1:**
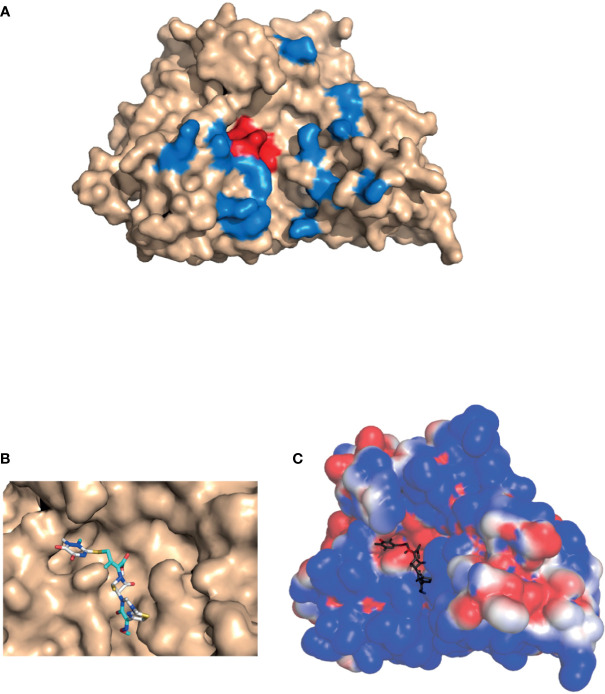
Artemis protein docking. 3-D structure of the catalytic domain of SNM1C/Artemis affinity with β-lactam antibiotics (PDB: 6WO0) (35). **(A)** Location of the β-lactamase conserved active site shown in red, and of the putative DNA-binding site in the pocket III (described by Karim *et al.* shown in blue. **(B)** Predicted binding of ceftriaxone to the SNM1C/Artemis target within its conserved active site. Molecular docking was performed using the AutoDockTools software. **(C) **Electrostatic potential surface of Artemis with predicted docking of ceftriaxone. An electrostatic potential surface of SNM1C/Artemis protein was generated using the PyMOL1.8.0 software along with the APBS tool plugin. The red color indicates an excess of negative charges near the surface and the blue color arises from a positively charged surface. The ceftriaxone scaffold is shown in dark grey.

SNM1C/Artemis, a 78 k-Da protein with 692 amino acids, is a member of the metallo-β-lactamase superfamily of nucleases, characterized by a conserved MBL and *β-CASP* domain. It belongs to a distinct group of the *β*-*CASP* family of nucleases that includes 3 proteins : SNM1A/Pso2 related proteins, SNM1B/Apollo, SNM1C/Artemis (38). The 3-dimensional structure of the catalytic domain of recombinant human SNM1C/Artemis has been recently solved (**Figure 1A**) (35). The SNM1C/Artemis’ active catalytic site contains metallo-β-lactamase, *β-CASP* domain and a cluster of conserved histidine and aspartate residues capable of binding two metals atoms. These particularities of the active catalytic site are similar to the other members of the DNA cross linked repair gene SNM1 family and mRNA 3’end processing endonuclease (35). As observed in the SNM1A protein, SNM1C/Artemis contains only one zinc ion in its catalytic site, the metallo-β-lactamase domain. The *β-CASP* domain acts as a plug, covering the active site displayed by the MBL domain, and creates the substrate binding pocket, which should confer substrate selectivity (35, 38). As particularities, the SNM1C/Artemis nuclease presents a second zinc ion in the *β-CASP* domain reorienting the putative DNA binding surface and extending the substrate binding pocket to a new pocket, the pocket III (**Figure 1A**) (35). This substrate binding site, pocket III, appears to be deeper and wider in the SNM1C/Artemis nuclease than in SNM1A and SNM1B proteins (35). In addition and contrary to the SNM1A and SNM1B proteins, SNM1C/Artemis exhibits a dominant and extensive positive charge in its substrate-binding surface, probably because of the structurally distinct DNA substrate of this protein (**Figure 1A**) (35). Mutation on the His-254 residue disrupts the *in vivo* and *in vitro* function of SNM1C/Artemis and results in the radiosensitive severe combined immune deficiency in human (see chapter V) (31, 35). His-254 residue is located within the catalytic metallo-β-lactamase domain and has been proposed to be involved at the metal ions coordination. It thus indicates that the unique zinc binding motif in the *β*-CASP domain of SNM1C/Artemis, which coordonate His-228, His-245, His 256 and Cys 272 residues, is both structurally and functionally important (35). Interestingly, the His-254 residue is conserved within the SNM1 family (31, 38).

### Artemis/SNM1C Protein and the V(J) and V(D)J Segments Rearrangement

**Figure 2:**
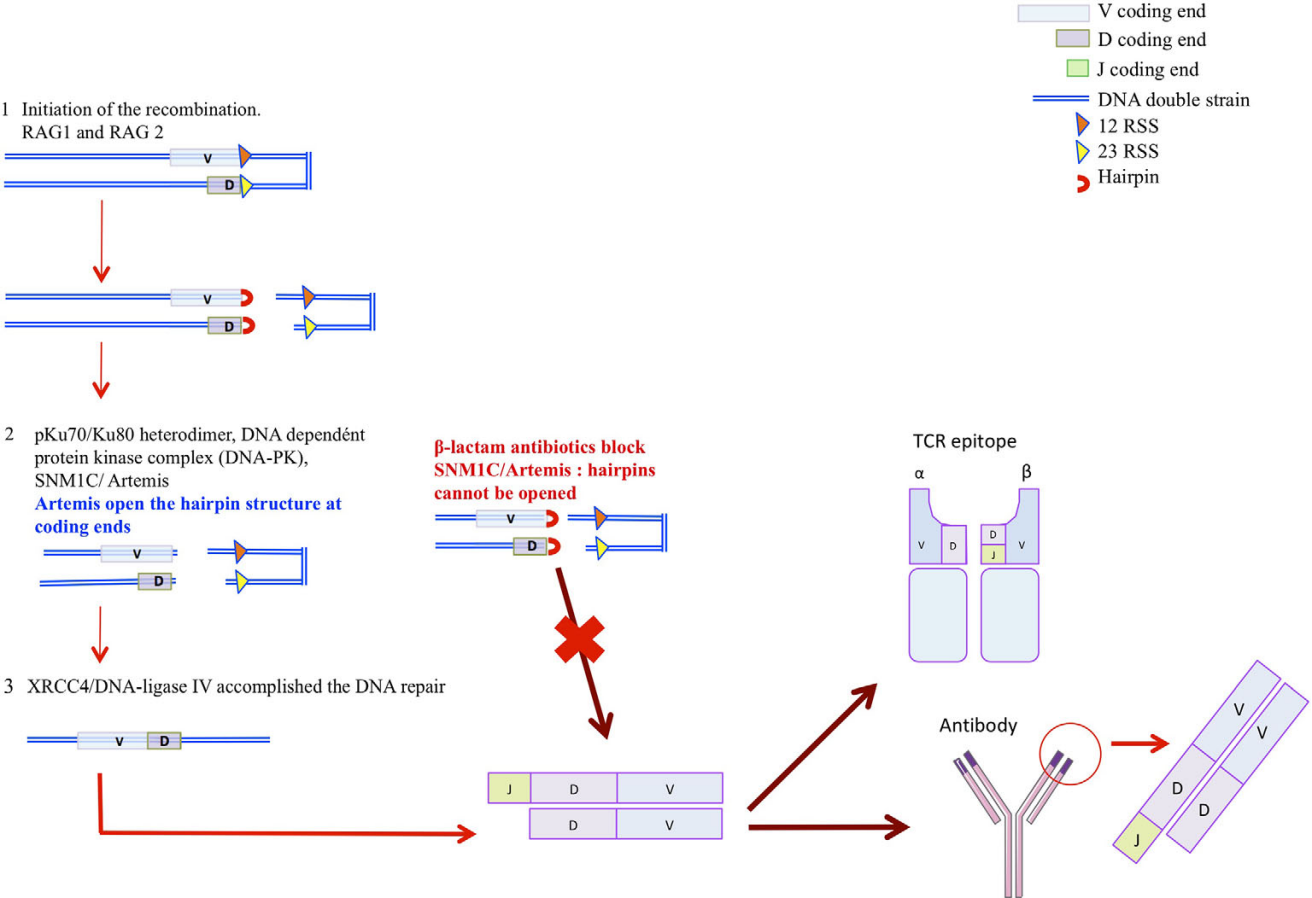
The hypothesis. 1-Lymphoid specific phase is the initiation of the recombination. RAG1 and RAG 2, recognize the recombination signal sequence (RSS) that flank all vairable (V), diversity (D), and joining (J) gene units and introduce the DNA double strand break (dsb) at the border of the RSS 2. This phase results on hairpin formation. 2-DNA damage is recognized and hairpin oppened, pKu70/Ku80 heterodimer bonds to the DNA dependant protein kinase complex (DNA-PK) that recognize the DNA dsb. Then the DNA-PKcs is recruited. DNA-PK recruits and phosphoryles SNM1C/Artemis protein that opens the hairpins structures at coding ends 3-DNA reparation is achieved since the XRCC4/DNA-ligase IV accomplished the DNA repai.

A central part of the immune system is played by antibodies that recognize and distinguish specific molecular patterns of antigens. Structure of antigens are different and the repertoire is currently estimated to be ~10^15^ members, for the naïve repertoire, and higher than 10^18^ based on the theorical repertoire combination (39). The variable region of immunoglobulins (VH and VL) and T-cell receptors (Vα, Vβ, V*γ*, and Vδ) are encoded by a modest region of gene segment whose diversity is achieved by a series of programmed DNA rearrangment termed the V(J) and V(D)J rearrangements (40–42). Immunoglobulin (Ig) or T-cell receptor variable regions are assembled in developping B or T lymphocytes from germline variable (V), diversity (D), and joining (J) gene units (42–44). While the V(D)J recombination segment is involved in the TCR β and δ and in the Ig heavy chain (IgH) variables exons, the V(J) recombination is involved in the TCRα and *γ* and Ig light chain (IgL) variables exons (43). These recombinations could be summarized in 3 different phases (**Figure 2**) (42, 43, 45, 46). The first one is lympoid specific and constitutes the initiation of the recombination. Two protein, RAG1 and RAG2, encode by the recombination activated gene 1 and 2 (RAG1 and RAG 2), recognize the recombination signal sequence (RSS) that flanks all variable (V), diversity (D), and joining (J) gene units and introduce the DNA double strand break (dsb) at the border of the RSS (45). Six proteins are involved in the second and third phases, which are constituted by the ubiquitous DNA-repair machinery (NHEJ), finalizing the DNA gap : Ku70/Ku80, DNA-dependent protein kinase catalytic subunit (DNA-PJcs), Artemis, terminal deoxynucleotidyl transferase (TdT) and XRCCA/LigaseIV (47). In the second phase, the DNA damage is recognized and hairpin are oppened. The pKu70/Ku80 heterodimer binds to the DNA dependent protein kinase complex (DNA-PK) that recognizes the DNA dsb. Then the DNA-PKcs is recruited and phosphoryles the SNM1/Artemis protein that opens the hairpins structures at coding ends (46). Finally, in the terciary phase, terminal deoxynucleotidyl transferase (TdT) ensures the jonctionnal diversity by adding N-nucleotides to the V-(D) and (D)-J jonction. DNA reparation is then achieved since the XRCC4/DNA-ligase IV accomplished the DNA repair. Ig and TCR diversity, is thus due to the inaccuracy in the junction areas V-(J) and V-(D)-J and to the insertion or deletion of a nucleotide under the action of the terminal transferase enzyme (TdT). If mutation in the RAG1/RAG2 compromises the initial phase of the V(D) and V(D)J recombination, defects in SNM1C/Artemis or in the DNA-PKcs induce a non-resolution of the hairpins at coding ends (**Figure 2**) (48). SNM1C/Artemis has thus a critical role on DNA double strand break repair in eukaryotic cells influencing the V(J) and V(D)J recombination and therefore the humoral and cellular immune diversity and functionality.

### SNM1C/Artemis Mutation in Humans

The Artemis autosomic recessive mutation induces default in the V(J) and V(D)J rearrangement causing humans RS-SCID phenotype characterized by an increase sensitivity to ϒ rays irradiation (**Figure 2**). SNM1C/Artemis gene mutation is rare in Europe, < 1/500 000 births, but its incidence is much higher in the Athabascan-speaking, Navajo and Apache Native Americans involving 1/2000 births (49). First cases of severe combined immunodeficiency were reported in Athabascan-speaking American Indian Children, who presented B-cells and T-cells deficiency associated with oral and/or genital ulceration (50). Clinical presentations were also characterized by hypogammaglobulinemia and poor antibody response (**Table 2**) (47, 49, 51, 52, 55–58). These children were dying in the primary year of life because of opportunistics infections caused by cytomegalovirus (CMV), Epstein barr virus (EBV), zooster virus (VZV), and pneumocystis. Reconstitution of the B and T lymphocyte pool was observed after bone marrow transplant therapy (BMT). Studying the prenatal diagnosis of severe combined immunodeficiency disease in the Athabascan population, Li et al. identified that 10% of unaffected carriers presented a polymorphism on the SCIDA mutation, located NspI exon 8 (59). In addition, hypomorphic Artemis mutant were reported as responsible for atypical severe combined immunodeficiencies complicated by autoimmunity, granulomatous inflammation, lymphoproliferative diseases and lymphoma malignancies with later clinical manifestations, in childhood or adulthood (37, 53, 58, 60). While the hMBLs SNM1C/Artemis mutations are associated with immunodeficiency, by analogy, reversible blockage of this protein may induce transient acquired immunodeficiency affecting the humoral response. The impact of cephalosporins on the immune response may have some similarities to the immune deficiencies seen in patients with an innate SNM1C/Artemis mutation. However, it is assumed that the impact of cephalosporins on the immune response is reversible and less severe than that seen in innate SNM1C/Artemis mutations. In the next chapter we will discuss the evidences arguing for a SNM1C/Artemis blockage by cephalosporins. In the next chapter we will discuss the evidences arguing for a SNM1C/Artemis blokage by cephalosporins.

**Table 2:** Clinical presentation of genetic SNM1C/Artemis mutation in human.

Mutation SNM1C/Artemis	Immune deficit	Clinical manifestations	Population	Ref
Deletion (exons 1–4, C279T; Genomic Deletion (exons 5–6)	B and T cell deficit	Early onset of severe infection	Athabascan-speaking, Navajo and Apache Native Americans	(47, 50)
Exon 11 splice donor (G→C); C279T		Severe oral and genital ulcer	Very high incidence: 1:2000	
Exon 10 splice donor (G→A)				
Exon 5 splice donor (G→T)				
Deletion (exons 1–4)				
Del G818				
Deletion (exons 5–8)				
Heterozygous and homozygous mutation (genomic exon 3 deletion; 2-nt insertion between T1205 and G1206)	Hypogammaglobulinemia	Otitis/diarrhea/stomatitis	2 & 3 months	(51)
Deletion exons 10, 11, and 12	B-cell differenciation arrest	Pneumocystis, CMV, VZV	1 day to 5 months	(52)
G>T mutation at position 47 in exon 5				
G>T mutation at position 47 in exon 5 homozygous G>A mutation at position 42 in exon 6				
Hypomorphic mutation (unprecised)	B and T cell lymphopenia and hypogammaglobulinemia	EBV associated disseminated B-cell lymphoma	NA	(53)
Hypomorphic DCLRE1C mutation	Antibody deficiency very low B-cell numbers and serum IgA levels	Recurrent respiratory tract infections, gastroenteritis, mycobacterial skin infection, otitis media, severe varicella, verruca vulgaris, aphtous stomatitis, granulomatous skin lesions, Hashimito thyroiditis, juvenile idiopathic arthritis	2-10 years	(54)
Hypomorphic DCLRE1C mutation	Chronic lymphopenia	Chronic ulcerating intestinal inflammation	6 years	(43)
Deletion in exon 14; c.1464delG; p.Gln488Fs	Hyper IgM syndrome, low IgG and IgA	Diarrhea, CMV, sclerosing cholangitis	6 & 5 year old	(55)
		Large granular lymphocytic leukemia, death		
Homologous deletion of the gene 3 of SNM1C/Artemis, DCLRE1C	NA	Pneumocystis	5 months	(56)
Mutation (c. 194C > T and c. 194C > T)	Poor antibody response	Chronic post vaccinal rubella virus infection (granulomatous dermatitis)	13 years	(57)

CMV, cytomegalovirus, VZV, varicella zooster virus, EBV, Epstein Barr virus, Ig, immunoglobulin; DCLRE1C, DNA, cross-link repair 1C.

## β-Lactam Antibiotics Can Inhibit the hMBLs SNM1 Fold

Some studies have demonstrated that β-lactam antibiotics inhibit the SNM1 (A, B & C) and *MBLAC1*.

First, *in vitro* experiment showed that ampicillin inhibit SNM1C/Artemis nuclease activity (33). Li et al. have evaluated the exo- and endo-nuclease activities of the SNM1C/Artemis protein in presence of ampicillin (33). Incubating the single-stranded DNA substrate with SNM1C/Artemis and ampicillin, they observed low levels of SNM1C/Artemis exonuclease and endonuclease products, in an ampicillin dose-dependent manner (33). Furthermore, as the active architecture site of bacterial MBLs and SNM1 proteins are similars, and as β-lactam antibiotics are the target of bacterial MBLs, Lee et al. screened the β-lactam antibiotics candidates inhibitors of SNM1A/B (32). Incubating 20 nucleotides single–stranded DNA substrates modified with fluorophore in the presence of the SNM1A/B proteins and cephalosporins, they observed, analysing the resultant fluorescent subtrate, that ceftriaxone acts as a competitive reversible inhibitors of the SNM1A/B protein (32). While the *in vitro* inhibitory concentration of ceftriaxone necessary to block SNM1A and SNM1B is 1mg/L, the plasmatic concentration reached in patients treated with ceftriaxone is much higher (32, 33). Interestingly and even more recently, a glial-expressed *Caenorhabditis elegans *gene, *swip-10*, an ortholog of the mammalian *MBLAC1* gene, that encodes for a metallo-β-lactamase domain-containing protein limiting glutamate-dependent changes in dopamine neuron excitability, showed high affinity with ceftriaxone (61). At last, using a real time fluorescence-based nuclease assay, ceftriaxone was identified as inhibiting the nuclease activity of the SNM1/C Artemis with a modest IC50 of 65 μM (34).

The ability for β-lactam antibiotics to interact with MBLs, whether in humans or other animals, was thus demonstrated *in vitro*. We will next review the *in silico* and *in vivo* evidences arguing for the possible SNM1C/Artemis inhibition by β-lactam antibiotics (**Table 3**).

**Table 3:** Evidences of the impact of β-lactam antibiotics on SNM1C/Artemis: *in vitro* and *in vivo* evidences of a decrease in the immune response especially the humoral response.

Molecule	Protein/Cell/animal/population	Administration	Evidence	Ref
***In vitro***				
Ceftriaxone	Metallo-β-lactamase domain-containing protein	High affinity of ceftriaxone to MBLAC1 catalytic site	Glial-expressed Caenorhabditis elegans gene, swip-10, encodes a metallo-β-lactamase domain-containing protein.	(61)
	MBLAC1		MBLAC1, a mammalian orthologue of swip-10	
			Ceftriaxone showed high affinity in binding to MBLAC1	
Cefotaxime	Resulting substrate fluorescence of SNM1A & B activity	SNM1A : IC_50_s of 4–7 μM	Competitive inhibitors of SNM1A and SNM1B exonuclease activity	(32)
Ceftriaxone		SNM1B : IC_50_s of 32–129 μM.		
Ampicillin	Evaluation of the SNM1C/Artemis products	5µM	Inhibition of the SNM1C/Artemis nucleases (endo & exo) activities	(33)
Ceftriaxone	SNM1C/Artemis	IC50 : 65 μM	Inhibition of the SNM1C/Artemis nuclease activity	(34)
**In human**				
Cephalosporin (within the last 3 months)	Adults	Intravenous	Increase risk of candidemia OR = 4, 95% CI 1.3-11 (p=0.01)	(62)
Cephalosporin (during ≥ 3 days)	Adults tertiary heath care hospital	Intravenous	Increase risk of candidemia (OR :2.2 IC [1.04-4.77] p=0.04) *versus* non peripheral line candidemia	(5)
Cephalosporin	Extremely low birth weight infants	Intravenous	Increase risk of candidemia (OR :1.77 95 IC[1,31-2,38] p<0.002)	(63)
Third-generation cephalosporin use	Neonate	Intravenous	Increase risk of candidemia OR: 4.6 (p <0.01)	(64)
**In animals**				
Ceftiofur hydrochloride	Pigs	Intravenous	Increase delay in the detection of pseudorabies virus vaccine antibodies	(65)
			Decrease antibodies levels in response to the swine influenza virus	
Intravenous cephalosporins (ceftizoxime, ceftezole and DQ2556)	Mice	Intravenous	Decrease response to pneumococcal type III (S3) polysaccharides	(66)
β-lactam cefsulodin, cefmenoxime, cefotiam and ceftazidime	Mice	Subcutaneously	Decrease IgG spleen level in mice treated with cephalosporins and immunized with red blood sheet cells than in those treated with penems	(67)
Ceftriaxone	Mice	Intragastric	Decrease B cells secreting antibodies in tissues (mesenteric lymph nodes, Peyer’s patches and GALTs)	(68)
			Decrease secreted IgA and sIgA	
Ceftriaxone	Mice	Daily gavage (150 days)	Decrease mucus IgG and IgA	(69)
			Spleen index reduction	

GALT, gut-associated lymphoid tissue; sIgA, secreted IgA; OR, Odd Ratio.

## *In Silico* Proof of Concept: Cephalosporin Can Interact With SNM1C/Artemis

In order to test our hypothesis we evaluate *in silico* the affinity of the SNM1C/Artemis protein with cephalosporin. The analysis showed a strong affinity of SNM1C/Artemis with cephalosporins predicting their possible interaction.

### Docking: Artemis Affinity With Cephalosporin

We performed a docking based on the three-dimensional structure of SNM1C/Artemis catalytic domain that was solved and published by Karim et al. (PDB: 6WO0) (35). They also described the pocket III structural feature of the SNM1C/Artemis as the putative DNA-binding site, that extends the conserved substrate-binding site and is made of mostly positively charged residues on the surface (**Figure 1A**). The interaction between the SNM1C/Artemis catalytic domain and the ceftriaxone β-lactam antibiotic could be modeled through molecular docking (**Figures 1B, C**) based on the previously mentioned structure (35). In our model, the cephalosporin appeared to preferentially bind within the conserved active site, mostly constituted of negatively charged residues on the surface, as shown with the electrostatic potential surface (**Figure 1C**). A binding energy of -3.84 kcal/mol could be predicted using the AutoDockTools software, suggesting promising affinity. This docking analysis is supported by a recent structural proposition, in which ceftriaxone binds to the SNM1C/Artemis protein surface (34).

## The *In Vivo* Impact of β-Lactam Antibiotics on Immunity, a Review of Evidences

Based on a review of the literature, we evaluate the evidences that β-lactam antibiotics have an impact on humoral immunity in animals and humans.

### β-Lactam Antibiotics Decrease the Humoral Response in Animals

Reports from veterinarians identified a decrease humoral response in animals treated with β-lactam antibiotics. In pigs, ceftiofur hydrochloride administrated concomitantly to vaccine induced a significant delay in the detection of antibodies in response to pseudorabies virus vaccine and a significant decrease of antibodies levels in response to the swine influenza virus, when compared to controls (65).

Furthermore, the immunotoxicity of intravenous cephalosporins (ceftizoxime, ceftezole and DQ2556) was demonstrated in mice, in which an increased spleen weight and enlargement of splenic germinal centres were observed 5 to 7 days after treatment (66). Cephalosporins are described as affecting cellular and humoral response (66). The response to pneumococcal type III (S3) polysaccharides, a T-cell independent immunogen, and the response to sheep red blood cells, a T-cell dependent immunogen, were decreased in mice treated 5 days with cephalosporins (66). In addition, the IgG level in the spleen of mice treated with cephalosporins and immunized with red blood cells was much lower than that of mice treated with penems (67). Finally, BALB/c mice treated with oral ceftriaxone showed a reduction in activated B and T cells in gut-associated lymphoid tissue (GALT), Peyer’s patches and mesenteric lymph nodes, as well as a significant reduction in mucus IgG and IgA (68, 69). Therefore, animals treated with cephalosporins were not able to mount a sustained protective immune response since a deficient humoral response was observed.

This may result, at least in part, on the blockage of the SNM1C/Artemis protein leading to reduced and delayed production of antibodies.

### Clinical and Experimental Reports in Human

In humans, several observations suggest the use of β-lactam antibiotics as a trigger for immunodeficiency, reducing the total number of leukocytes, promoting opportunistic infections and decreasing the response to anticancer therapy with immune checkpoint inhibitors.

In a retrospective analysis of 291 patients treated with antibiotics, leukopenia in addition to neutropenia, thrombopenia and eosinophilia were identified as adverses events of parenteral antibiotics use (70). Interestingly, leukopenia was mainly observed in patients receiving β-lactam (39%) as the only antibiotic (70).

Antibiotics may also facilitate opportunistic infections commonly encountered in immunocompromised patients since the use of β-lactam antibiotics has been reported to be significantly associated with an increased risk of candidemia in neonates, infants and adults (**Table 3**) (5, 60, 62, 63). If the importance of the humoral response in the control of candidiasis has been discussed, and is supported by several *in vitro* and *in vivo* observations (71–74). Anti MP-65 antibodies were related with survival in patients with severe disseminated infection and the administration of human polyvalent IgG mouthwash showed significant reduction of infection in patients with chronic oral candidiasis (62, 71).

In cancer patients, the use of antibiotics before treatment with immune checkpoint inhibitors is significantly associated with relapse and worse clinical outcome (8, 75, 76). Several types of cancer are concerned: renal cell carcinoma, non small cell lung cancer, urothelial carcinoma and melanoma (8, 75, 76). Interestingly, all authors of these studies agreed that β-lactam antibiotics were the most widely used antibiotics (35 to 45%) followed by quinolones and macrolides (8, 75, 77). It would have been interesting to explore the lymphocyte pool of cancer’s patients receiving ceftriaxone to test our hypothesis.

It should be noted that the direct impact of cephalosporin on B-cells is probably not the only risk factor that could explain the occurrence of candida infection since catheters, microbiota composition and TH17 cell response are clearly involved in these infections (5, 63, 78, 79). Nonetheless, these *in vivo* observations support the possible immunomodulatory and even immunosuppressive role of β-lactam antibiotics on immune cells favouring the occurrence of opportunistic infections.

## Discussion

### β-Lactam Antibiotics May Block the SNM1C/Artemis, hMBLs, Activity

**Figure 3:**
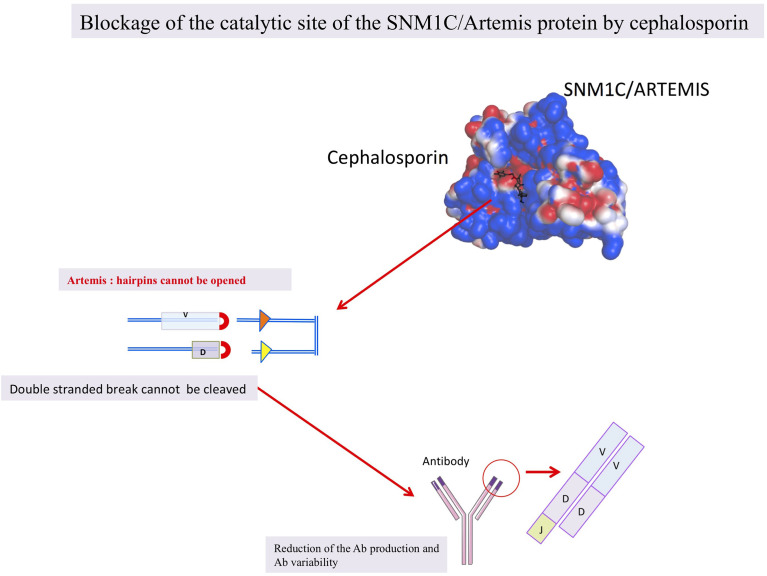
The hypothesis shematized: β-lactam antibiotics blocking the SNM1C/Artemis protein.

We postulate that β-lactam antibiotics can induce reversible humoral deficiency in humans by transiently blocking the SNM1C/Artemis nuclease activity and our hypothesis is sustained by *in silico*, *in vitro* and *in vivo* observations (**Figure 3** and **Table 3**) (33, 35). β-lactam antibiotics may act reversibly on the hMBLs, SNM1C/Artemis protein, blocking V(J) and V(D)J recombination resulting in a deficient humoral responses. Therefore, the clinical immunocompromized stage observed under β-lactam antibiotics could be linked to the SNM1C/Artemis blockage. Shedding light on this issue would make it possible to improve care. Indeed, if β-lactam antibiotics have an impact on immunity, diminishing the host’s ability to mount a strong vaccinal response, a delay between antibiotic administration and vaccination should be proposed based on further investigations. Moreover, if β-lactam antibiotics confer immunocompromised states and favour opportunistic infections, candidal infections should be actively sought and routinely reported in the event of fever under these antibiotics, the use of catheters should, in addition, be limited.

### Further Evidences Are Needed to Confirm This Hypothesis

*In vitro* experimentations are needed to demonstrate that β-lactam antibiotics, such as cephalosporin, may block the SNM1C/Artemis protein inhibiting the substrates production of this exo and endonuclease. *In vivo*, testing the ability of mice to produce an effective vaccine response when treated with ceftriaxone, in addition to the evaluation of their B and T cell maturated pool will provide answers to these questions. In humans on cephalosporin, humoral immunity can be studied simply by assessing the vaccine response. Further research evaluating the humoral response, in particular against *C. albicans*, in patients receiving β-lactam antibiotics, would shed light on our hypothesis. We have focused here on hMBLs but MBLs are present in the world of life, in bacteria, eukaryotes and viruses, so the spectrum of β-lactam may be broader than expected and may also involve some MBLs.

### Metallo β-Lactamase in the World of Life

**Figure 4:**
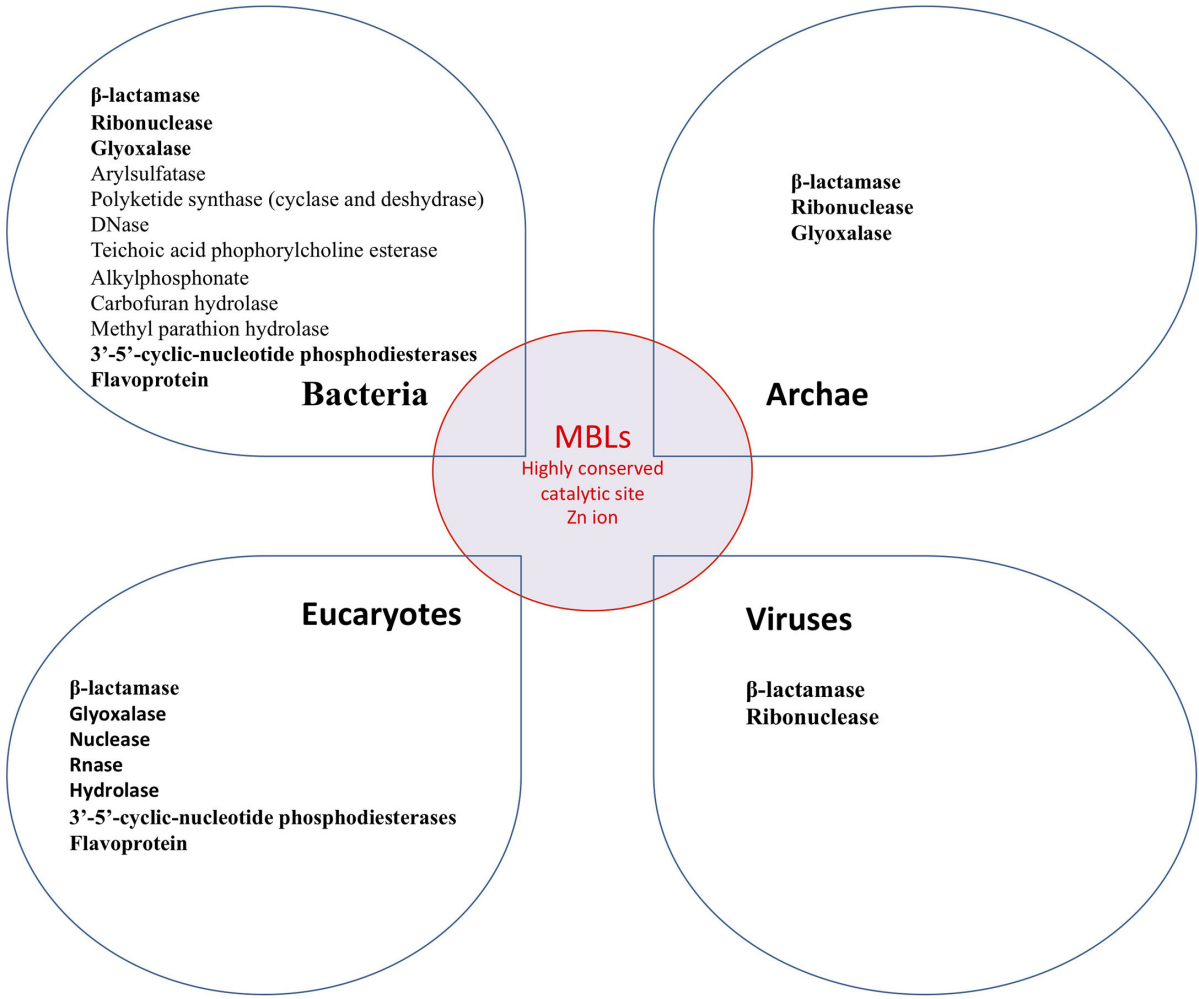
Known MBLs enzymatic activity in the world of life. All these enzymes may be inhibited by β-lactam antibiotics.

It is important to point out that MBLs are widely distributed in nature. They are found in bacteria, archae, giant viruses, and eukaryotes cells including humans. Thus a landscape of cross enzymatic activities among MBLs enzyme superfamily was reported (31, 77, 80–83). Therefore, MBLs’ cross enzymatic activities (hydrolase and nuclease) among different domains of the living were identified, whereas different enzymatic activities observed may be related to divergent host substrates (17). The interspecies common enzymatic activities can be explained by the highly conserved MBLs catalytic site (**Figure 4**) (81, 84, 85). Thus, while the catalytic site of MBL is highly conserved, all known enzymatic activities of MBL could possibly be inhibited by β-lactam antibiotics (**Figure 3**). In humans, blocking the enzymatic activities not only of the SNM1C/Artemis protein but of each of these 18 hMBLs may have a physiological and clinical relevance as yet unknown.

### Microbiota and the Indirect Role of β-Lactam on the Immune Response, a Confounding Marker

While our hypothesis supports the direct role of β-lactam on immune cells, the impact of these antibiotics *via* the microbiota on immunity - *i.e.* their indirect impact on the immune response - cannot be neglected. In animals, a reduced antibody vaccinal response was observed in germ-free, antibiotic-induced dysbiosis and antibiotic-treated mice. Interestingly, these antibody vaccinal responses were re-increased after oral reconstitution of the gut microbiota (9–11). In humans, the role of the gut microbiota was also highly suspected to modulate the vaccinal response. *Bifidobacterium* predominance enhanced humoral response to oral and parenteral vaccine (oral polio vaccine, Bacille calmette guerin vaccine, tetanus toxoid) (12, 14, 15). High abundance of *Proteobacteria, Clostridium cluster XI*, and *Streptococcus bovis* and low abundance of *Bacteroidetes* phylum were associated with an increase serological response to oral rotavirus vaccine (12, 14, 15). More recently, a reduced H1N1 vaccinal (IgG and IgA) response was reported in patients treated with neomycin, vancomycin and metronidazole (13). Thus, although the impact of β-lactam on vaccine response has not been specifically studied, antibiotics appear to modulate the humoral response to vaccination, and probably, in part, *via* the gut microbiota.

## Conclusion

β-lactam antibiotics direct impacts on the immune cells should be considered. Since antibiotics can inhibit SNM1A, B and C/Artemis activities *in vitro*, and since the catalytic site of hMBL, in particular that of the SNM1 family, is very well conserved, we hypothesized that β-lactam antibiotics could reversibly inhibit the SNM1C/Artemis protein and block the V(D)J recombination. Our hypothesis is sustained by *in vitro*, *in silico* and *in vivo* evidences and needs to be further investigated. The putative interference of beta-lactams with SNM1C/Artemis nuclease activity would become clinically relevant in cases of long-term treatment, such as persistent osteoarticular, cardiovascular or cerebro-meningeal infections. Highlighting the physiological role and clinical impact of the hMBLs as the effect of β-lactam antibiotics on these hMBLs could pave the way for new clinical and therapeutic approaches in immunology, infectious diseases and oncology.

## Data Availability Statement

The raw data supporting the conclusions of this article will be made available by the authors, without undue reservation.

## Author Contributions

CM and PP wrote the article. LP performed the in silico analysis. CD, J-LM, and DR supervised the manuscript. All authors contributed to the article and approved the submitted version.

## Funding

URMITE, IHU Méditerranée Infection. This work was supported by the French Government under the « Investissements d’avenir » (Investments for the Future) programme managed by the Agence Nationale de la Recherche (ANR, fr: National Agency for Research), (reference: Méditerranée Infection 10-IAHU-03). This work was also supported by Région Provence-Alpes-Côte d’Azur and European funding FEDER PRIMMI (Fonds Européen de Développement Régional - Plateformes de Recherche et d’Innovation Mutualisées Méditerranée Infection.

## Conflict of Interest

The authors declare that the research was conducted in the absence of any commercial or financial relationships that could be construed as a potential conflict of interest.

## Publisher’s Note

All claims expressed in this article are solely those of the authors and do not necessarily represent those of their affiliated organizations, or those of the publisher, the editors and the reviewers. Any product that may be evaluated in this article, or claim that may be made by its manufacturer, is not guaranteed or endorsed by the publisher.
